# Information and Communication Technologies for Managing Frailty: A Systematic Literature Review

**DOI:** 10.14336/AD.2020.1114

**Published:** 2021-06-01

**Authors:** Antonio Miguel Cruz, Laura Monsalve, Anna-Maria Ladurner, Luisa Fernanda Jaime, Daniel Wang, Daniel Alejandro Quiroga

**Affiliations:** ^1^Department of Occupational Therapy, Faculty of Rehabilitation Medicine, University of Alberta, Edmonton, AB, Canada.; ^2^Glenrose Rehabilitation Research, Innovation & Technology (GRRIT) Hub, Glenrose Rehabilitation Hospital, Edmonton, AB, Canada.; ^3^Faculty of Applied Health Sciences, University of Waterloo, Waterloo, ON, Canada.; ^4^School of Medicine and Health Sciences, Universidad del Rosario, Bogota, Colombia.

**Keywords:** Information and telecommunication technologies, frail, fragility, frail older adults

## Abstract

Frailty is a prevalent condition among Canadians; over one million are diagnosed as medically frail, and in the next ten years this number will double. Information and telecommunication technologies can provide a low-cost method for managing frailty more proactively. This study aims to examine the range and extent of information and telecommunication technologies for managing frailty in older adults, their technology readiness level, the evidence, and the associated outcomes. A systematic literature review was conducted. Four databases were searched for studies: Medline, EMBASE, CINAHL, and Web of Science. In total, we included 19 studies (out of 9,930) for the data abstraction. Overall, our findings indicate that (1) the proposed frailty phenotype is the most common ground truth to be used for assessing frailty; (2) the most common uses of information and telecommunication technologies for managing frailty are detection, and monitoring and detection, while interventional studies on frailty are very rare; (3) the five main types of information and telecommunication technologies for managing frailty in older adults are information and telecommunication technology-based platforms, smartphones, telemonitoring (home monitoring), wearable sensors and devices (commercial off-the-shelf), and multimedia formats for online access; (4) the technology readiness level of information and telecommunication technologies for managing frailty in older adults is the “Technology Demonstration” level, i.e., not yet ready to be operated in an actual operating environment; and (5) the level of evidence is still low for information and telecommunication technology studies that manage frailty in older adults. In conclusion, information and telecommunication technologies for managing frailty in the older adult population are not yet ready to be full-fledged technologies for this purpose.

Over one million Canadians are diagnosed as medically frail, and in the next ten years the number of Canadians living with frailty will double (Available from: http://www.cfn-nce.ca/frailty-in-canada/). Studies have shown that frailty scores increase nonlinearly with age, the risk of mortality increases with frailty scores, and women have higher frailty scores than men by as much as 100 % [1]. Increases in frailty scores have a strong association with adverse health outcomes (e.g., chronic non-communicable diseases) and healthcare [1].

Frailty is studied using either one of two approaches, i.e., the *phenotypic approach* [2] and the *deficit accumulation approach* [3] (see the Key definitions section for more details). Irrespective of the approach, frailty screening is conducted on a non-routine basis in primary care or clinical settings [4]; as a result, "hidden health vulnerabilities" cannot be identified early enough and, therefore, the prevention of frailty is not possible. A new approach to frailty is needed that relies on regular and systematic monitoring to support preventive interventions [5]. We believe that ICT can provide a low-cost method for managing frailty more proactively, i.e., measuring, monitoring, and preventing frailty in the older adult population continuously and in their homes. Our premise takes on special importance in the wake of the COVID-19 pandemic in particular, as this new situation has affected those aged 65 years and older [6]. Many initiatives have been implemented in order to monitor patients in their homes [7-8], although monitoring frailty at home is a topic that appears to be lagging behind. For example, the report entitled Promoting Healthy Ageing through a Frailty Prevention Approach (FPA) across Europe, is the result of a comprehensive initiative which aimed to determine the state of the art on frailty and to create a rationale for a common European framework for a frailty prevention and management approach. In this report, ten domains for the comprehensive management of frailty were established (Available from: www.advantageja.eu/images/FPA-Core-ADVANTAGE-doc.pdf). Domain nine specifically highlights the need to implement support systems (i.e., finance and information and communication technologies (ICT)) to “be able to measure, monitor and report important measures of frailty outcomes in different settings including all determinants of health”. Unfortunately, only a third of European Union countries report the use of ICT solutions to prevent or manage frailty, and only 15% of them allocate economic resources to enhance the implementation of a national/regional strategy on frailty prevention. Some literature reviews about the use of ICT for managing frailty in the older adult population have been published. Dasenbrock, Heinks, Schwenk, & Bauer [9] conducted a literature review that focused on describing the potential of information and sensor technology to objectively assess the functionality and mobility of pre-frail and frail older adults in a variety of settings, namely, clinical settings and everyday life. In another literature review, Mugueta-Aguinaga & Garcia-Zapirain [10] examined what technological resources (i.e., devices and programs) are used to manage frailty, specifically those related to prevention, diagnosis, care, and treatment. Selak, Bacaicoa, & Gabrovec [11] conducted a narrative review that aimed to explore the effects of ICT that are used to support the management of frail people. Finally, Gallucci, et al. [12] conducted a systematic literature review that aimed to synthesize the current evidence on the use of ICT for managing frailty care in older adults. One of the limitations of the study by Gallucci and colleagues was that they limited their selection to systematic literature reviews published between 2015 and the present. In doing so, their study was biased as it was prevented from having a more comprehensive view of the research on ICT for managing frail older people.

In fact, only two systematic literature reviews were included in their qualitative synthesis. Another limitation of Gallucci and colleagues' study was the inclusion and synthesis in their results of studies from the two selected reviews. In this way, Gallucci and colleagues may have incorporated and perpetuated any pre-existing bias that existed in these two reviews. Another limitation of all the previous literature reviews was their lack of any clear definitions of some of the crucial terms used, such as ICT, chronic conditions or diseases, and frailty management, thus causing readers to be unclear about the boundaries of the applications of ICT for managing frailty. To sum up, the four aforementioned literature reviews analyzed a total of 239 studies, all of which report that very few studies strictly focused on frailty as an outcome variable, and the interventional study approach on frailty using ICT was almost non-existent. In order to fill these gaps, we want to expand on the previous literature reviews with the specific purpose of examining the range and extent of ICT for managing frailty in older adults, their technology readiness level, the evidence, and the associated outcomes. The specific research questions of this literature review are

1) What ground truths are used to measure frailty in studies that implement ICT solutions?
2) What types of ICT are used to manage frailty in older adults, and to what extent are they used?
3) What technology readiness level are ICT for managing frailty in older adults at?
4) What are the outcomes and variables when ICT are used to manage frailty in older adults?
5) What level of evidence is there for ICT interventions for managing frailty in older adults?

## ICT, technology readiness level, chronic conditions or diseases, frailty, and disease and frailty management: Key definitions

This literature review is driven by the concepts of ICT, the ICT technology readiness level (TRL), chronic conditions or diseases, frailty, and disease and frailty management. ICT have been defined as “a set of activities and technologies that fall into the union of IT [Information Technology] and telecommunications” (Available from: www.ets.org/Media/Research/pdf/ICTREPORT.pdf.), whereas Zhang and colleagues [13] define ICT as “technologies used by people and organizations for their information processing and communication purposes” (p. 628). In the context of this systematic literature review, we will use the term ICT as defined by the Information Technology Infrastructure Library (ITL), i.e., “the application of science to the processing of data according to programmed instructions in order to derive results. In the widest sense, the ICT refer to all networking components, applications, devices, and systems that allow you to connect with the digital world” (Available from: www.techsling.com/what-is-ict-and-how-does-it-fit-within-itil/). Overall, the technology readiness level indicates the maturity of a given technology[14], in our case ICT. According to the United States Department of Energy, the TRL scale ranges from 1 (lowest TRL, basic principles observed) through 9 (highest TRL, the total system used successfully in project operations). The literature reports a variety of ICT that are used to manage the medical conditions of frail older adults with, namely, ICT-based platforms, smartphones, telemonitoring (home monitoring), wearable sensors and devices (commercial off-the-shelf (COTS), and multimedia formats for online access for a diverse range of applications including activity recognition, behavioral pattern discovery, abnormal activity detection or anomaly detection, planning and scheduling, and decision support [15-16]. A COTS can be defined as “a commercial item sold in substantial quantities in the commercial marketplace; and is offered to the Government, under a contract or subcontract at any tier, without modification, in the same form in which it is sold in the commercial marketplace” (Available from: www.law.cornell.edu/cfr/text/48/2.101).

According to the Public Health Agency of Canada (Available from: https://cbpp-pcpe.phac-aspc.gc.ca/chronic-diseases/#:~:text=Chronic%20diseases%2C%20also%20known%20as,be%20treated%20but%20not%20cured.), chronic diseases, also known as non-communicable diseases (NCDs), are persistent and generally slow in progression, and they can be treated but not cured. Frailty is understood as being a state of increased vulnerability, with reduced physical reserves and loss of function in multiple body systems [2]. Frailty has been studied using either one of two approaches, i.e., the phenotypic approach [2] and the deficit accumulation approach [3]. The former approach operationalizes frailty as poor performance in three out of the five following criteria: weight loss, exhaustion, weakness, slowness, and lack of activity. The latter approach operationalizes this condition using the frailty index, i.e., the number of deficits that a person exhibits out of a list of deficits included in the frailty index estimation instrument. The deficit accumulation approach, operationalized via the frailty index, is a number between 0 and 1. In this systematic literature review, we use the definition of disease management (DM) provided by [17], i.e., “interventions to prevent or manage one or more chronic conditions, with the objective to identify persons at risk for one chronic condition, to promote self-management by patients, and to achieve the best clinical outcomes”. Extrapolating the concept of DM to frailty management (FM), we understand FM as being a systematic population-based approach developed for screening and monitoring (to identify the frailty level), prevention (to promote self-management by people with pre-frailty conditions), and treating or delaying frailty (interventions and measurement of clinical outcomes).

## METHODS

### Design

In this systematic literature review, we followed five steps: (1) we formulated the research questions based on the *PICOS* guidelines (Population, Intervention, Comparison, Outcome, Study type); (2) searched the databases to identify the relevant studies; (3) selected the studies according to the inclusion criteria; (4) charted the data; (5) and collated, summarized, and reported the results [18-19]. This systematic literature review follows the Preferred Reporting Items for Systematic Reviews and Meta-Analyses (PRISMA) reporting guidelines [20].

### Information sources

We searched the following four electronic databases: Medline (Version: OVID MEDLINE(R) 1946 to present), EMBASE (Version: OVID Embase 1974 to present), CINAHL (Version: CINAHL Plus with Full Text), and Web of Science (Version: Web of Science Core Collection). The search took place from February 25, 2020 to February 26, 2020.

### Search strategy

We used the following concepts to search the electronic databases and extract the studies: Concept 1 (frailty), Concept 2 (ICT), and Concept 3 (older adults). The keywords for these concepts were used in combination with the “*AND”* and “*OR*” Boolean operators (see Supplementary Table 1). A librarian with expertise in the area of health sciences supervised and approved the search strategy conducted by the members of the team.

### Inclusion criteria

(1) Studies that included ICT that:
  a Addressed technology used for measuring, monitoring, or managing frailty in home or supportive care environments for adults, regardless of whether the technology was embedded in the building structure or worn on the person with a frailty.
  b Have been implemented or deployed at least in pilot form (i.e., ICT technology readiness level (TRL) ≥ 4 (according to the [[14])) with a minimum of one adult as a participant with a focus on measuring, monitoring, or managing frailty for adults.
(2) Studies that included adult participants aged 65 years or older.
(3) Studies published in Spanish, English, or German available in full text in peer-reviewed journals or conference proceedings from electronic abstract systems.
(4) Studies that used any study design or methodology, with positive or negative results.
(5) Studies that used any approach for measuring frailty (i.e., the phenotype or commutative index approach).

### Exclusion criteria

We excluded the following:

(1) Studies published before 2010.
(2) Studies published in books, book chapters, and Ph.D. or Master’s theses.
(2) Papers that were lecture notes at conferences, theoretical/seminal papers, narrative reviews, meta-analyses, and other types of literature review.
(2) Research conducted in hospitals or rehabilitation facilities.
(2) Papers that did not provide enough information in order to categorize them (e.g., description of the participants, technology readiness).
(2) Abstracts or papers that were not available.
(2) Papers that only assessed the usability, acceptance, and/or technology adoption of ICT for managing frailty.
(2) Papers that only used telephone interventions (e.g., a nurse calling a patient to ask what the patient had done) or any other kinds of interventions for managing frailty with no other technology (e.g., no sensors).
(2) Study protocols.
(2) Any kind of electronic Frailty Index (eFI) that was collected or calculated from electronic health records (EHRs).
(2) Papers that only focused on one dimension of a frailty measurement (e.g., timed up and go with sensors).
(2) Beyond the scope.

### Study selection process

For the selection phase, we followed a variation of the procedures in Neubauer, et al. [21] and Miguel-Cruz, et al. 21514 [19]. First, two members of the research team (AML and LM) exported all of the identified studies to the EndNote X7.7.1 ? (1988-2016 Thompson Reuters) reference manager software. One member of the research team (AML) removed any duplicates. Second, prior to the title and abstract evaluation phase, every member of the team (AMC, LM, AML, LFJ, DW, and DAQ) was trained in how to apply the inclusion and exclusion criteria. Third, two pairs of independent researchers (pair 1: AMC and AML; pair 2: LM and LFJ) evaluated the titles and abstracts of the remaining studies and compared them with the inclusion and exclusion criteria. Any differences between the two pairs of independent researchers with regard to deciding whether to include a study in the next phase were addressed in a meeting where each pair of researchers discussed the differences independently. If there was still disagreement about the suitability of a study’s abstract, one individual from the other pair of researchers acted as a third rater in order to make a final decision about whether to include that study in the full reading study phase (e.g., AMC acted as a third rater for disagreements between pair 2, LM and LFJ). Fourth, the same two pairs of researchers (i.e., pair 1: AMC and AML; pair 2: LM and LFJ) who evaluated the title and abstract phase reviewed the full texts of the selected studies. A third rater made the final decision about whether to include these studies in the data extraction phase (e.g., DW or DAQ).

### Data extraction process

During this phase, two pairs of independent researchers (pair 1: AMC and AML; pair 2: LM and LFJ) completed the data extraction on the final selected papers and annotated the operationalization of the variables in an Excel spreadsheet file. Both pairs of researchers met to reconcile any differences in the data extraction through discussion. If there was any disagreement about the extracted information, one of the researchers acted as a third rater to make a final decision about what information to include in the spreadsheet Excel file. The research team ensured that this third rater did not review the same papers that he/she had performed the data extraction on. Each selected study was reviewed, and the data were extracted for this study’s domains of interest.

### Data analysis

One member of the team (DAC) conducted the data analysis under the supervision of the first author (AMC). The studies were categorized into three main groups according to the main goal of the ICT and study goals, as follows: (1) detection of frailty, (2) detection of and monitoring frailty, and (3) the interventional or clinical study. In this literature review, detection is understood to be the action or process of identifying the presence of something concealed, whereas monitoring is the action of “observing” the progress or quality of (something) over a period of time. In our case, what is being detected and/or monitored is frailty or another variable as a proxy (e.g., physical activity). Finally, a clinical study is understood to be a study that aims to evaluate the impact of ICT on frailty as an outcome variable that used any methodological approach (qualitative, quantitative, or mixed). It is understood that studies that aim to detect and/or monitor frailty are not interventional. We assessed the quality of the quantitative studies for randomized controlled trial (RCT) (if any) using the PEDro scale [22] and the strength of the evidence using an adaptation of the modified Sackett criteria (Available from: https://pdfs.semanticscholar.org/2b08/beaff788b361f6e3f5ca37ed8fda616b3fa9.pdf). According to Teasell’s approach, evidence is assessed on levels ranging from conflicting evidence to Level 1a, the highest level of evidence (Supplementary Table 2). We used descriptive statistics to characterize the studies included in our literature review. In some cases, we allowed data such as diagnoses to be counted manifold.

**Figure 1.**
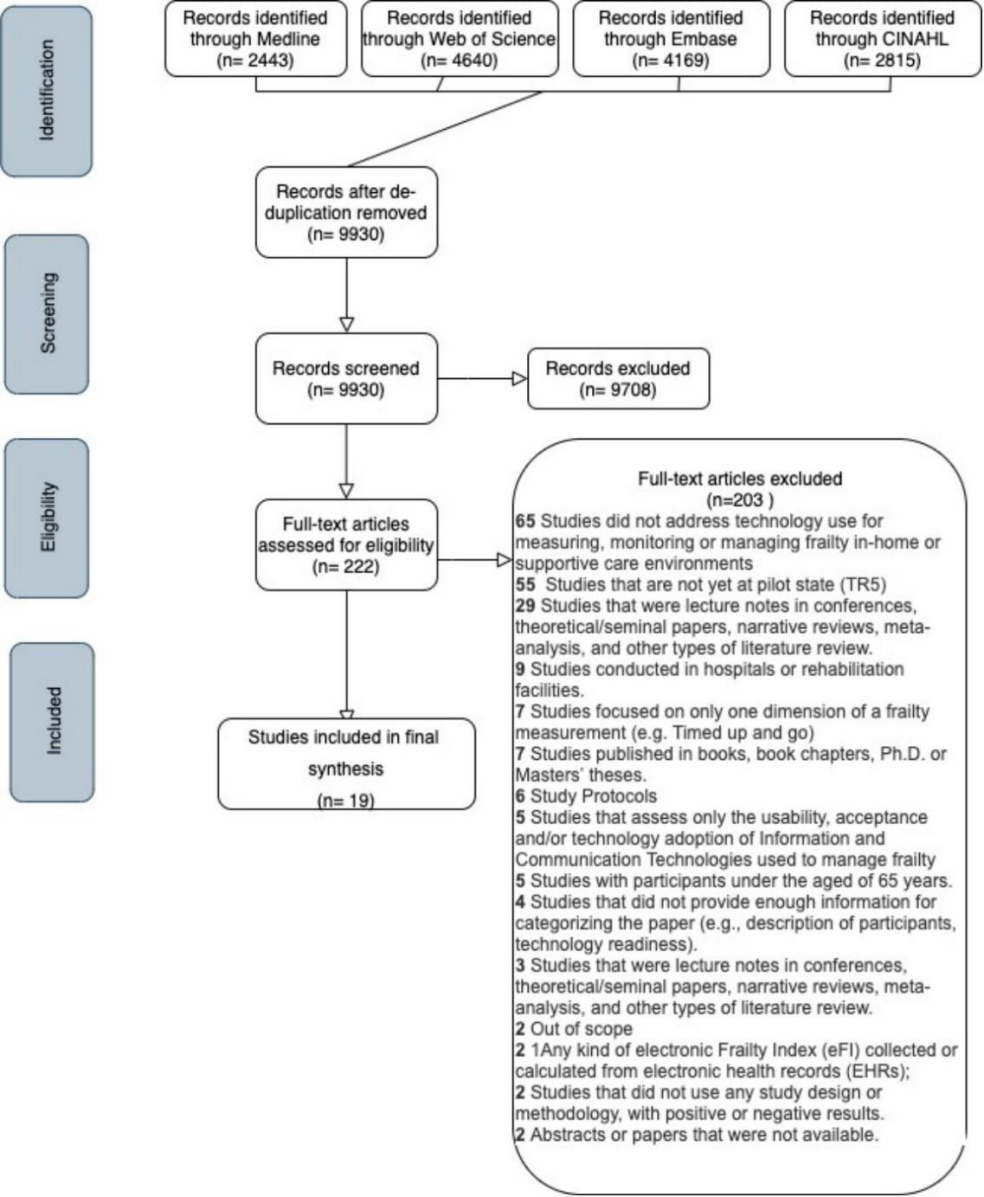
Scholarly reviewed literature article search results.

## RESULTS

### Study selection

Figure 1 shows the results of the review process. We identified 14,067 studies; after removing duplicates, a total of 9,930 (70.6%, 9,930/14,067) studies remained for the title and abstract screening phase. We excluded 9,708 (97.8%, 9,708 /9,930) studies during this phase. During the full-text reading phase, we excluded 203 studies. In total, we included 19 studies for the data abstraction. The level of agreement between the raters during both the title and abstract phase and the full-text screening phase was high, with a 93.53% level of agreement for the abstracts and 91.2% for the full papers.

### Population

Overall, the studies included 5,337 (range 17 to 1,527) subjects in total. The participants were older adults (67% women) with an average age of 76.83 (SD=5.14) years, and a mean age ranging between 65 and 83.7 years. Although the majority of the medical conditions were not reported, most of the participants had heart and chronic obstructive pulmonary disease (COPD) medical conditions (see Table 1 for a detailed characterization of the population).

### Settings

The studies were mainly conducted at the participants’ homes (68.4%, 13/19). The other recruitment settings were a long-term facility (5.3%, 1/19), a community care center (5.3%, 1/19), and other mixed settings (15.8%, 3/19), e.g., home and nursing homes (5.3%, 1/19), home and long-term settings (5.3%, 1/19), and home and city (5.3%, 1/19). In one case, the settings were not reported (5.3%, 1/19) (see Table 3 for a detailed characterization of the included studies).

### Study goals and designs

The main purposes of the relevant studies included just detection (47.4%, 9/19), and monitoring and detection (42.1%, 8/19)) of frailty. There were only two interventional studies on frailty (10.5%, 2/19). The most common study designs were case study and correlational study designs (73.7%, 14/19). The correlational study designs aimed to detect and/or monitor frailty variables using ICT. Then, in order to test the validity of the data collection methods using ICT, the authors correlated these data with established frailty assessing tools (e.g., the Fried frailty phenotype scale). The other studies’ designs included cross-sectional (15.8%, 3/19) and descriptive ones (5.3%, 1/19) (see Tables 2 and 3 for a detailed characterization of the included studies).

**Table 1:** Population characteristics and frailty level.

Study type	n (%)	Population					
		Sample size (N)	Sex Female % Male %	Age (mean (SD))	Medical condition	Frailty level [average, or categories] (number of studies)	References
Detection	9 (47)	801	71% 29 %	78.23 (7.1)	NR (8) NA (1)	Non-frail, pre-frail, frail (5) Frail (2) NR (2)	[27-35]
Detection and monitoring	8 (42)	4,421	55% 45%	79.29 (3.16)	Hypertension (2) NR (2) Congestive heart failure (CHF) (1) Chronic obstructive pulmonary disease (COPD) (1) Heart attack (1) Hypercholesterolemia (1)	Frail, robust (2) Pre-frail, frail (1) Frail, fit (1) Frail, not-frail (1) Pre-frail (1) Frail, very frail (1) Frail (1)	[5, 36-42]
Interventional study	2 (11)	115	66% 34%	70.59 (3.80)	NR (2)	SOF Score (1) Pre-frail, robust (1)	[43-44]

NR: Not reported; NA: Not applicable

**Table 2:** Study design and types of ICT used.

Study type	n (%)	Study design and goals and type of ICT used								
		Study design (number of studies)	Quantitative study design (number of studies)	Settings (number of studies)	Length of the study (weeks) mean (SD)	Level of evidence	Frailty instrument used (number of times used)	TRL mean (SD)	Overall, ICT type used (number of studies)	References
Detection	9 (47)	Quantitative (9)	Case study design (3) Correlational study (3) Cross-sectional Design/Survey based (1) Descriptive (1) Cross-sectional descriptive (1)	Home (7) Long-term facility (1) Home and long-term facility (1)	18.36 (28.60)	Level 5	NR (5) Fried frailty phenotype scale (3) Frailty modeled through activity performance (1)	5.88 (0.33)	Telemonitoring (home monitoring) (4) Wearable sensors (3) Smartphone (1) Telemonitoring (home monitoring) & wearable sensors (1)	[27-35]
Detection and monitoring	8 (42)	Quantitative (8)	Case study design (5) Correlational study (3)	Home (6) Home and city (1) NR (1)	56.66 (63.71)	Level 5	Fried frailty phenotype scale (3) Clinical Frailty Scale (CFS) (1) Groningen Frailty Indicator (GFI) (1) Tilburg Frailty Indicator (1) Edmonton Frailty Scale (1) NR (1)	5.8 (0.57)	Telemonitoring (home monitoring) & wearable sensors (2) Wearable sensors (2) Telemonitoring (home monitoring) (2) ICT-based platform (1) Smartphone (1)	[5, 36-42]
Interventional study	2 (11)	Quantitative (2)	Cross-sectional Design/Survey based (1) Pre and post-experimental group and control (not randomized) (1)	Home (1) Community care center (1)	44.0 (16.97)	Level 5	Study of Osteoporotic Fracture index (SOF) (1) Cardiovascular Health Study (CHS) frailty phenotype criteria (1) Clinical Frailty Scale (CFS) (1)	7 (0)	Multimedia format for online access and smartphone (1) Wearable device & smartphone (1)	[43-44]

NR: Not reported; NA: Not applicable

### Ground truths used for measuring frailty in studies that implement ICT solutions

The frailty phenotype proposed by Fried, et al. [23] was the most common ground truth to be used for measuring frailty in the included studies (31.6%, 6/19). Two studies (10.5%) used the Clinical Frailty Scale (CFS) designed by Mitnitski and Rockwood [24]. Other less commonly used scales were the Study of Osteoporotic Fracture index (SOF) (5.3%, 1/19), frailty status modeled through activity performance (5.3%, 1/19), the Cardiovascular Health Study (CHS) frailty phenotype criteria (5.3%, 1/19), the Edmonton Frailty Scale [25] (5.3%, 1/19), and the Tilburg Frailty Indicator [26] (5.3%, 1/19). In six studies (31.6%, 6/19) there was no information about a specific frailty model.

The ground truths used for measuring frailty in studies that implement ICT solutions were stratified in some cases. For example, five studies (26.3% , 5/19) stratified the participants into three levels of frailty (non-frail, pre-frail, and frail), seven studies considered two levels of frailty, that is, frail, robust (10.5%, 2/19); pre-frail, frail (5.3%, 1/19); frail, fit (5.3%, 1/19); frail, not-frail (5.3%, 1/19); pre-frail, robust (10.5%, 2/19); and frail, very frail (5.3%, 1/19). Four studies considered participants without any frailty stratification (21.1%, 4/19), i.e., three studies only considered frail participants (15.8%, 3/19), whereas one study (5.3%, 1/19) only considered pre-frail participants. In two cases, the frailty stratification was not reported (10.5%, 2/19) (see Tables 2 and 3 for a detailed characterization of the included studies).

**Figure 2.**
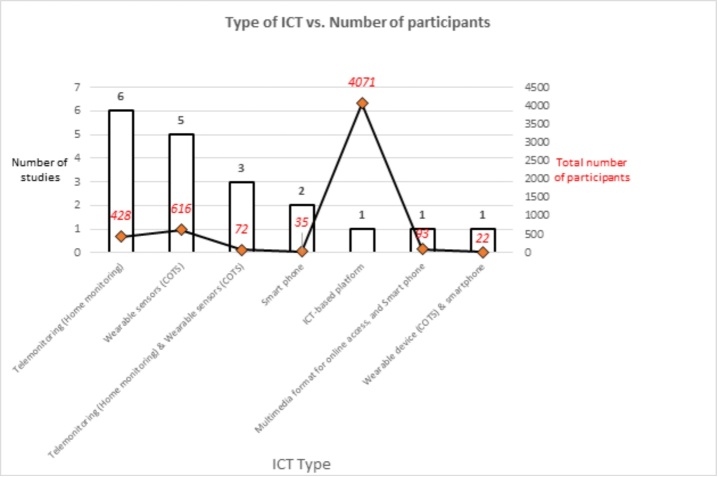
Types of ICT vs. Number of participants.

### ICT for managing frailty in older adults

Overall, the studies included in this literature review used five main types of ICT to manage frailty in older adults, namely, ICT-based platforms, smartphones, telemonitoring (home monitoring), wearable sensors and devices (commercial off-the-shelf (COTS)), and multimedia formats for online access. These five main types of ICT were used either alone or in combination to manage frailty in older adults. Telemonitoring (home monitoring) (31.6%, 6/19), wearable sensors (COTS) (26.3%, 5/19), smartphones (10.5%, 2/19), and ICT-based platforms (5.3%, 1/19) were used alone. In contrast, wearable devices (COTS) and smartphones (5.3%, 1/19), multimedia formats for online access and smartphones (5.3%, 1/19), and telemonitoring (home monitoring) and wearable sensors (COTS) (15.8%, 3/19) were used in combination (see Table 3 and Figure 2 for a detailed characterization of the ICT used in the included studies).

At a more granular level, in studies where telemonitoring (home monitoring) ICT were employed, sensors such as non-invasive blood pressure sensors, oxygen saturation (SpO2) sensors, weight sensors, passive infrared (PIR) motion sensors, pressure sensors installed in beds and chairs, electrical sensors installed in doors and/or cabinets, ultrasonic distance sensors, and Bluetooth Beacons (Non Line Of Sight environments) were the most commonly used. When smartphones (COTS) and/or wearable sensors (COTS) were used to detect and/or monitor frailty, tri-axial accelerometers, magnetometers, gyroscopes, near-field communication (NFC) tags, microphones, X-Band Doppler Motion Detectors, grip-balls (with incorporated pressure sensors), and doppler sensors were used (see Table 3 for a detailed characterization of the ICT used in the included studies).

### Technology readiness level of the ICT used to manage frailty in older adults

Overall, the technology readiness level of the ICT used to manage frailty in older adults was 6.00 SD (0.57), meaning these technologies are at the “*Technology Demonstration*” level [14]. In other words, the ICT used to manage frailty in older adults are at the prototype stage. At this stage, the technology is capable of performing every function that will be required in an actual operating environment (e.g., the homes of older adults), but the developing and testing (e.g., the final design is now virtually complete) phases of the technology are not yet ready for operation in an actual operating environment. We compared the technology readiness level of the ICT used to manage frailty in older adults in interventional and non-interventional studies (i.e., detecting and monitoring frailty). We found statistically significant differences between the TRLs of the ICT used in two groups [F(1, 17) = 10.09, p = 0.006], (mean TRL _interventional studies_= 7.00.SD (0.00), mean TRL _non-interventional studies_= 5.88 SD (0.48)). We also found a statistically significant positive correlation between the types of studies and the TRLs of the ICTs, i.e., the ICT used in interventional studies are at higher levels of technology readiness r_xy_ (17) = +0.610, p=0.006.

**Table 3:** Characteristics of the included studies.

References and study type	Sample size (N)	Sex (Female = %)	Mean age (SD)	Frailty level used (average, or categories)	Frailty instrument used	Design type (quantitative)	Settings	Length of the study (weeks)	Name of the system/platform	TRL	ICT used	Sensors/devices
Detection [35]	20	Female= 50%	83.7 (NR)	Frail	NR	Descriptive	Long-term facility	6	No name	6	Smart phone (COTS)	Tri-axial accelerometer
Detection [27]	125	Female= 75.0%	>65 years (NR)	Non-frail, pre-frail, frail	Fried frailty phenotype scale	Cross-sectional descriptive	Home	56	No name	6	Wearable sensors (COTS)	LEGSys and BalanSens. Five inertial sensors (A triaxial accelerometer, magnetometer, and gyroscope PAMSys. Inertial sensors (triaxial accelerometer)
Detection [28]	119	Female=79%	77.465 (7.4)	Non-frail, pre-frail, frail	Fried frailty phenotype scale	Correlational study	Home and long-term	0.28	No name	5	Wearable sensors (COTS)	PAMSys
Detection [29]	8	NR	NR (NR)	NR	NR	Correlational study	Home	NR	No name	6	Telemonitoring (home monitoring)	Pressure sensor, ultrasound distance sensor
Detection [33]	73	Females= 65.7%	78.15 (5.5)	Non-frail, pre-frail, frail	NR	Correlational study	Home	1	No name	6	Telemonitoring (home monitoring)	Bluetooth Beacons (Non Line Of Sight environments)
Detection [30]	271	Females=62.4%	76.8 (5.3)	Non-frail, pre-frail, frail	NR	Case study design	Home	1	No name	6	Telemonitoring (home monitoring)	Bluetooth Beacons (Non Line Of Sight environments)
Detection [34]	8	NR	NR (NR)	Frail	Frailty modeled through activity performance	Case study design	Home	64	UbiSMART AAL platform	6	Telemonitoring (home monitoring)	Industrial sensors
Detection [31]	153	Female= 79%	75 (10)	Frail, non-frail, pre-frail	Fried frailty phenotype scale	Cross-sectional Design/Survey based	Home	0.28	Pendant sensor	6	Wearable sensors (COTS)	Pendant sensor (PAMSys) (three-dimensional accelerations)
Detection [32]	24	Female= 62.5%	>65 years (NR)	NR	NR	Case study design	Home	NR	City4Age	6	Telemonitoring (home monitoring) *AND* wearable sensors (COTS)	Prototypal wristband (9-axis inertial sensors), paired with a smartphone, smartphone’s GPS interface
Detection and monitoring [5]	4071	NR	>65 years (NR)	Pre-frail and frail	Groningen Frailty Indicator (GFI)	Case study design	Home	156	PERSSILAA.	5	ICT-based platform	Smartphone, mobile and home sensing devices, and step counter
Detection and monitoring [38]	15	Female= 66.66%	75.3 (1.8)	Frail, fit	Fried frailty phenotype scale	Correlational study	Home	NR	InCense	6	Smartphone (COTS)	Smartphone, accelerometer, GPS, near-field communication (NFC) tags, microphone
Detection and monitoring [39]	194	Female= 59.8%	78.9 (5.7)	Frail, not-frail	Fried frailty phenotype scale	Correlational study	NR	NR	ARPEGE Pack	6	Wearable sensors (COTS)	X-Band Doppler Motion Detector MDU 1130, bathroom scale, balance quality tester, grip-ball, doppler sensor, and tablets
Detection and monitoring [40]	45	Female= 32%	79.51 (NR)	Frail, robust	CSHA Clinical Frailty Scale	Case study design	Home and city	95	No name	6	Telemonitoring (home monitoring) *AND* wearable sensors (COTS)	Prototypal wristband (9-axis inertial sensors), paired with a smartphone, smartphone’s GPS interface
Detection and monitoring [36]	3	Female= 66.66%	86.7 (3.5)	Pre-frail	NR	Case study design	Home	12	Fragil-IT	6	Telemonitoring (home monitoring) *AND* wearable sensors (COTS)	Walking radar, bathroom scale, infrared sensors (indoor), insole (outdoor), dynamometer, tablet
Detection and monitoring [41]	25	Female= 60%	71 (6)	Frail, robust	Tilburg Frailty Indicator	Correlational study	Home	1	ADAMO	7	Wearable sensors (COTS)	Care-watch
Detection and monitoring [37]	36	Female= 62%	82 (10)	Frail, very frail	Edmonton Frailty Scale	Case study design	Home	52	No name	6	Telemonitoring (home monitoring)	BP, blood pressure sensor, SpO2, weight, PIR motion sensor, bed sensor (pressure sensors), chair sensor (pressure sensors)
Monitoring and managing [42]	32	Female=75%	81.63 (1.6)	Frail	Fried frailty phenotype scale	Case study design	Home	24	HomeAssist	5	Telemonitoring (home monitoring)	Wireless sensors, sensors in doors
Interventional study [43]	22	Female= 8%	70.591 (3.801)	Robust, pre-frail	Cardiovascular Health Study (CHS) frailty phenotype criteria CSHA Clinical Frailty Scale	Cross-sectional Design/Survey based	Home	56	No name	7	Wearable device (COTS) and smartphone	Xiao Mi band 2. Sensors in a wearable device did not provide details
Interventional study [44]	93	Female= 73.1%	65 (NR)	SOF score Mean=1.82	Three items from the Study of Osteoporotic Fracture index (SOF)	Pre and post-experimental group and control (not randomized)	Community care center	32	No name	7	Multimedia format for online access, and smartphone	NA

NR: Not reported; NA: Not applicable

### The outcome variables and study results when ICT are used to manage frailty in older adults

Overall, a total of 29 outcome variables were used. The most common outcome variables used to detect, monitor, and/or improve frailty were physical activity (34.5%, 10/29), postural balance (17.2%, 5/29), gait speed (13.8%, 4/29), grip strength (10.3%, 3/29), the European Quality of Life-5 Dimensions (6.8%, 2/29), well-being (6.8%, 2/29), sleep quality (6.8%, 2/29), activities of daily living (ADLs) (6.8%, 2/29), and instrumental activities of daily living (IADLs) (6.8%, 2/29).

In the studies about frailty detection, a more specific aim was to employ ICT to measure once (cross-sectional) the frailty components by using variables such as gait speed, postural balance, and/or physical activity. Then, for validity purposes the authors correlated these measurements with the frailty scales as a ground truth (e.g., the Fried frailty phenotype scale). In this regard, while in some studies where ICT were used along with algorithms they were able to discriminate (detect) between different frailty levels (e.g., three levels, i.e., frail, non-frail, pre-frail) [27-32], other studies either failed to detect frailty [33] or did not provide any quantitative data about the accuracy of the algorithms used or the correlation coefficients [34-35].

In the studies about frailty detection and monitoring, the outcome variables were measured continuously. Unlike the studies related to the frailty detection group, in the frailty detection and monitoring group the aim was to employ ICT to continually assess and monitor the evolution of older adults with regard to their health status, instrumental activities of daily living, well-being, quality of life-5, sleep quality, etc., and then to use those variables to conduct a frailty assessment, mostly in the form of two categories (e.g., frail, robust; frail, not-frail; or frail, very frail). Most of the studies in this group were descriptive. As in the frailty detection group studies, some studies asserted that the ICT employed could be used for screening and preventing frailty; however, no quantitative data were provided [5, 36-37], whereas other studies were found to monitor the evolution of the monitored variables (e.g., subject activity and frailty status, [38-42]) effectively.

Two studies were found in the interventional study category. One study aimed to determine whether the Smart Walk program could increase physical activity and the health outcomes of small groups of older adults in rural areas [43]. The Smart Walk program was a 13-month program consisting of six months of coaching management, one month of rest, and then six months of self-management. The participants all wore a wearable device. In order to quantitatively evaluate the participants’ frailty status, the authors used a frailty index, i.e., the Clinical Frailty Scale (CFS), as the primary outcome variable. This intervention effectively improved physical fitness, anthropometric measurements, and geriatric assessment categories (Clinical Frailty Scale (CFS)) in a small group of older adults from rural areas with limited monitoring resources. The second study aimed to explore the effects (on reversing frailty and improving health) of a health promotion program on community-dwelling middle-aged and older adults [44]. The intervention consisted in training material for improving balance and 20-minute flexibility exercises, although the authors did not specify how many times per week or per day. The outcome variable was three items for frailty from the SOF index. This study showed that the frailty scores improved significantly in the experimental group compared to the control (p-value < 0.00).

### Level of evidence for ICT studies that manage frailty in older adults

Based on the findings from the case and correlational study designs, there is *Level 5* evidence that ICT can detect the frailty status of older adults when the following variables are used: postural balance, sleep quality, activities of daily living, gait speed, temporal-spatial gait parameters, reaction times, slowness of movement, physical performance, motility, and instrumental activities of daily living. Based on the findings from the case and correlational study designs, there is *conflicting* evidence that ICT can detect frailty in older adults when the physical activity outcome variable is used.[Table T4-ad-12-3-914]

**Table 4 T4-ad-12-3-914:** Outcomes and variables used in ICT studies for managing frailty details.

Study type	n (%)	Results and outcome variables				
		Frailty instrument used (number of times used)	Outcome variables (number of times used)	References	Outcome variable details	Results
Detection	9 (47%)	Frailty modeled through activity performance (1) Fried frailty phenotype scale (3) NR (5)	Physical activity (6) Postural balance (3) Sleep quality (2) Activities of daily living (2) Gait speed (1) Temporal-spatial gait parameters (1) Reaction time (1) Slowness of movement (1) Physical performance (1) Motility (1) Instrumental activities of daily living (1)	[35]	Physical activity Gait speed Postural balance	Purpose: To develop a system to support physicians in determining an accurate and centralized elderly frailty diagnosis. Results: No conclusive results were obtained. At the time of publication the system was in evaluation, testing different kinds of patients by using the developed method.
				[27]	Physical activity Temporal-spatial gait parameters Postural balance	Purpose: To examine the ability of wearable sensor-based in-home assessment of gait, balance, and physical activity to discriminate between frailty levels (non-frail, pre-frail, and frail). Results: The system was able to discriminate (detect) between three frailty levels. Gait parameters were found to be the most sensitive for the identification of a subject’s level of frailty.
				[28]	Physical activity	Purpose: To explore the use of daily postural transition quantified using a chest-worn wearable technology to identify frailty in community-dwelling older adults. Results: The system effectively identified the daily number of specific postural transitions such as walk-to-stand and quick sitting could be used for monitoring frailty status.
				[29]	Slowness of movement Postural balance Reaction time	Purpose: To develop a wireless home-based frailty detection system. Results: The proposed system was effective at detecting frailty, i.e., there were close correlations between the standard instruments for measuring frailty and the measures of frailty using the proposed system.
				[33]	Physical activity (room-to-room transitions)	Purpose: To develop a system based on the analysis of data describing daily in-house activities for the assessment of frailty in older people, Results: The system failed to classify the three-class frailty status, with a maximum accuracy of 59.06%
				[30]	Physical activity (room-to-room transitions)	Purpose: To develop an indoor localization system for monitoring the mobility behavior of older individuals and to assess the correlation between the measured indoor activities of an older person and his/her frailty status. Results: The system effectively identified the frailty status of older adults, i.e., it had an accuracy of 83% in the classification of a monitored person regarding his/her frailty status (frail, pre-frail, non-frail).
				[34]	Physical performance Activities of daily living Sleep quality	Purpose: To detect frailty levels using the UbiSMART system. Results: The system was able to characterize the frailty dimensions for a given senior person on a given day, although no quantitative data are provided.
				[31]	Physical activity Sleep quality	Purpose: To determine whether a pendant accelerometer device in the home setting is sensitive to identifying pre-frailty. Results: The pendant sensor could effectively identify pre-frailty via daily home monitoring (i.e., the high area under the curve 0.88, meaning high discrimination power).
				[32]	Motility Activities of daily living Instrumental activities of daily living	Purpose: To develop a critical performance analysis of an IoT-aware Ambient Assisted Living system for monitoring the elderly. Results: The results obtained in the analysis demonstrate the generally satisfactory forecasting performance and therefore validate the overall usefulness of the data-driven forecasting approach (average error percentage 13.10%).
Detection and monitoring	8 (42)	Clinical Frailty Scale (CFS) (1) Fried frailty phenotype scale (3) Groningen Frailty Indicator (GFI) (1) Tilburg Frailty Indicator (1) Edmonton Frailty Scale (1) NR (1)	Physical activity (3) Grip-strength (2) Gait speed (2) European Quality of Life-5 Dimensions (1) Disability (1) Falls (1) Institutionalization (1) Hospitalization (1) Mortality (1) Behavior data (1) Weight (1) Exhaustion (1) Behavioral changes (1) Postural balance (1) Mobility Index (1) Health status (1) Well-being (1) Caregiver burden (1) Instrumental activities of daily living (1)	[5]	European Quality of Life-5 Dimensions Disability Falls Institutionalization Hospitalization Mortality	Purpose: To develop an ICT platform (PERSSILAA) to screen, assess, manage, and monitor pre-frail community-dwelling older adults in order to address pre-frailty and promote active and healthy aging. Results: Twenty-five healthcare-related recommendations from PERSSILAA were provided, exploring how they could be used in the development of future European guidelines on the screening and prevention of frailty. No data about the specific effectiveness of the PERSSILAA platform in reducing the frailty levels or quality of life of older adults are provided
				[38]	Physical activity Behaviour data	Purpose: To determine whether a mobile phone can be used to approximate the amount of physical activity performed by an older adult, thus better understanding their mobility patterns and assessing aspects related to frailty. Results: The data obtained from the mobile phone allowed the doctor’s assessment and diagnosis/prognosis of frailty to be supported.
				[39]	Physical activity Weight Grip-strength Gait speed Exhaustion	Purpose: To determine whether the data produced by a technological set (ARPEGE Pack) are equivalent to those obtained by usual clinical tests, as well as to discuss whether the ARPEGE Pack can be used for remote long-term frailty monitoring. Results: Correlations regarding weight, grip strength, and walking speed confirm the validity of the data produced by the ARPEGE Pack to feed Fried’s criteria (r= 0.89).
				[40]	Behavioral changes (outdoor walking distance, weekly visits pattern)	Purpose: To describe a longitudinal cohort study in smart cities for assessing early frailty symptoms while deploying an unobtrusive IoT-based system. Results: Analysis of the collected data enabled the absence of frailty (robust or post-robust status) to be identified.
				[36]	Physical activity Grip-strength Gait speed Postural balance	Purpose: To develop home monitoring (Fragil-IT) to assess and monitor the evolution of older adults’ state of health based on a physical frailty assessment. Results: The Fragil-IT system provided instrumented data to doctors in order to monitor the evolution of subject activity, and to deduce the effects of prescribed medicines. No quantitative data are provided.
				[41]	Mobility index	Purpose: To evaluate differences in the mobility index (MI) provided by an innovative remote monitoring device (ADAMO) for older adults and to compare the association of the MI and a traditional physical measure with frailty. Results: ADAMO was able to measure mobility level information about individual health status and specifically about frailty.
				[37]	Health status Well-being	Purpose: To investigate the potential of an integrated care system that acquires data on vital clinical signs and habits to support independent living for elderly people with chronic diseases. Results: Our results suggest that integrated care monitoring technologies have the potential to provide improved care and could have a positive impact on the well-being of the elderly by enabling timely interventions. No data on the effectiveness of the system are provided.
				[42]	Caregiver burden Instrumental activities of daily living	Purpose: To assess the benefits of a multi-task Ambient assisted living (AAL) platform for both Frail older Individuals (FIs) and professional caregivers with respect to everyday functioning and caregiver burdens. Results: A reduction in self-reported objective burdens was obtained after six months of AAL.
Interventional study	2 (11)	Study of Osteoporotic Fracture index (SOF) (1) Cardiovascular Health Study (CHS) frailty phenotype criteria (1) Clinical Frailty Scale (CFS) (1)	SOF index (1) Grip strength (1) Postural balance (1) Flexibility (1) Well-being (1) Gait speed (1) Physical activity (1) European Quality of Life-5 Dimensions (1)	[44]	Three items for frailty from the SOF index Well-being.	Purpose: To explore the effects (on reversing frailty and improving health) of a health promotion program on community-dwelling middle-aged and older adults. Results: Exercise intensity, exercise duration, and frailty scores significantly improved in experimental group over the control;
				[43]	Physical activity Postural balance Flexibility European Quality of Life-5 Dimensions Grip strength	Purpose: To evaluate whether a wearable device and mobile-based intermittent coaching or self-management (Smart Walk program) could increase physical activity and health outcomes of small groups of older adults in rural areas. Results: The “Smart Walk” program improved physical fitness, anthropometric measurements, and geriatric assessment categories (Clinical Frailty Scale (CFS)) in a small group of older adults in a rural area with limited resources for monitoring.

NR: Not reported; NA: Not applicable

Based on the findings from the case and correlational study designs, there is *Level 5* evidence that ICT can detect and monitor the frailty status of older adults when the following outcome variables are used: physical activity, grip strength, gait speed, the European Quality of Life-5 Dimensions, disability, falls, institutionalization, hospitalization, mortality, behavior data, weight, exhaustion, behavioral changes, postural balance, mobility index, health status, well-being, and instrumental activities of daily living.

Based on the findings from a pre and post-experimental group and control (not randomized) study design [43], there is *Level 4* evidence that ICT (i.e., wearable devices and mobile-based intermittent coaching or self-management (Smart Walk program)) have an effect on the related frailty components including physical activity, postural balance, flexibility, and grip strength in frail older adults.

Based on the findings from a cross-sectional design/survey-based study design [44], there is *Level 5* evidence that ICT (i.e., an IT platform and a cellphone-based health promotion program) have an effect on reversing frailty (measured using the SOF index) in older adults.

## DISCUSSION

Our systematic literature review aims to examine the range and extent of ICT for managing frailty in older adults, their technology readiness level, the evidence, and the associated outcomes. By exploring the technology readiness level of ICT in particular and its evidence in clinical studies, our systematic literature review has produced new knowledge compared to previous literature reviews that explored the use of ICT for managing frailty in older adults [9-11]. We included 19 studies (out of 9,930) and, overall, our findings indicate that (1) the frailty phenotype proposed by Fried, et al. [23] was the most common ground truth to be used for assessing frailty; (2) the most common uses of ICT for managing frailty were detection, and monitoring and detection, while interventional studies on frailty were very rare; (3) the five main types of ICT technologies for managing frailty in older adults were ICT-based platforms, smartphones, telemonitoring (home monitoring), wearable sensors and devices (commercial off-the-shelf (COTS)), and multimedia formats for online access; (4); the technology readiness level of ICT for managing frailty in older adults was at the “Technology Demonstration” level, i.e., not yet ready to be operated in an actual operating environment; and (5) the level of evidence was still low for ICT studies on managing frailty in older adults.

We found, as in Gallucci and colleagues’ [12] systematic literature review, the frailty phenotype approach proposed by Fried, et al. [23] was the most common ground truth to be used for assessing frailty symptoms. We also found that the deficit accumulation approach was only used on two occasions (i.e., [36, 43]), and those studies that used the frailty phenotype approach did not consider all five core components. Different explanations could account for this result. First, for practical reasons, the frailty phenotype scale is shorter and can be easily instrumented by means of ICT (e.g., COTS dynamometers, sensors, wearable sensors (COTS), etc.). In contrast, a more comprehensive assessment of frailty, such as CSHA Clinical Frailty, which follows the logic of the deficit accumulation approach, requires different ICT to be integrated. This is because at least 30 frailty components should be measured in order to assess the frailty deficit accumulation approach accurately [45], thus resulting in more costly and long-lasting research studies. Second, from the data analysis point of view, the frailty phenotype approach can be stratified into two or three levels of frailty (e.g., non-frail, pre-frail, frail; or frail, not-frail), whereas the frailty index is a continuous number between 0 and 1, thus making it more difficult to stratify the frailty index. By stratifying the frailty phenotype approach, the frailty level is easier to detect because it becomes a “classification problem”, whereas treating it as a continuum means that in order to be detected it requires the application of other models such as linear regression, thus demanding larger sample sizes.

We found the most common ICT for managing frailty were detection, and monitoring and detection, while interventional or clinical studies on frailty were very rare. This explains, in turn, why we only found one study where frailty was used as a truly outcome variable [44]. This result did not surprise us, as this agrees with Kuchel [4]’s findings, i.e., few published clinical trials have included frailty as an outcome measure. The fact that most of the studies were related to the use of ICT for screening and monitoring instead of prevention, treating, or delaying frailty, can be explained according to the point of view or findings of the Promoting Healthy Ageing through a Frailty Prevention Approach (FPA) report and the State of the Art Report on Frailty (SoAR)(Available from: www.advantageja.eu/images/FPA-Core-ADVANTAGE-doc.pdf). The SoAR report showed that European Union Member States are classified as ‘basic’(i.e., nothing is going on in European Union Member States in relation to that item) in terms of population screening, and monitoring and surveillance of frailty (Available from: www.advantageja.eu/images/FPA-Core-ADVANTAGE-doc.pdf). It is quite apparent that as the first priority has been to identify the population group at the highest risk of frailty, ICT development goes hand in hand with this priority. Unfortunately, as the use of ICT to deal with the problem of frailty has followed a reactive approach (detection, and monitoring and detection) instead of a proactive one, the opportunities that ICT are giving us to test the effects of interventions on frailty prevention or modification within our lifetimes are being missed. In short, we will have to wait in order to observe a greater penetration and use of ICT in preventing, treating, or delaying frailty.

The fact that we only found two interventional studies on frailty can be explained by one of our findings, i.e., the technology readiness level of ICT for managing frailty in older adults is not yet ready to be operated in an actual operating environment. The low technology readiness level of ICT for managing frailty could lead to another problem, i.e., low levels of usability and technology acceptance. This aspect creates a barrier against the successful implementation of ICT for managing frailty [46]. For example, a recent literature review that aimed to survey the use of technology in the management of frailty concluded that, “further work needs to be carried out to reduce the gap existing between technology, frail older adults, healthcare professionals, and carers to bring together the different views about the use of technology”[47].

We found that most of our outcome variables that were used in ICT studies on frailty were oriented toward dealing with the problem of screening (detecting) and monitoring frailty, i.e., physical activity, postural balance, and gait speed, while very few were used to tackle the problem of diagnosing, treating, or delaying frailty. This finding can be explained for a couple of reasons. One possible explanation is that the requirement of ICT to measure frailty in a more comprehensive way such as the use of the frailty index of accumulative deficits is more complicated, as this requires a more diverse range of more complex, more expensive, and higher risk medical equipment (e.g., electrocardiography monitors). Another possible explanation is that it is quite apparent that if all “eyes are fixed” in terms of population screening, monitoring, and surveillance of frailty, the implementation of ICT for frailty is oriented toward the use of commercial off-the-shelf solutions. The reason this happens is because with this range of equipment, screening and monitoring frailty is easier, more sustainable, and cheaper.

We found the level of evidence for studies on ICT for managing frailty in older adults is still low. This low level of evidence hinders significant progress in clinical frailty prevention, as it leads to reluctance on the part of health professionals and clients (older adults) toward the use of ICT. Studies have found that the most critical factor in the implementation and acceptance of healthcare technologies is the demonstrated level of evidence regarding effectiveness in achieving clinical outcome goals [48]. Therefore, the implementation of studies such as RCT on ICT for managing frailty in older adults should be implemented in the short term in order to achieve high levels of evidence.

The design and implementation of evidence-based practice, such as the use of ICT for managing frailty depend on successful behavioral change interventions [49]. Surprisingly, only one study has explicitly used behavioral changes, which were measured using outdoor walking distances and weekly visiting patterns as outcome variables [40]; more importantly, none of the studies identified in this literature review used a theoretical approach regarding behavioral change. One of these frameworks is the well-known 'COM-B' system. In this system, capability, opportunity, and motivation interact to generate behavior that, in turn, influences these components. The theoretical underpinnings of this model are that a particular behavior will only occur when a person has the capability and opportunity to engage in that behavior and is motivated enough to enact it [49]. We believe that future interventions that are designed to manage frailty through the use of ICT should take into account existing frameworks based on the model of behavior, i.e., behavioral change interventions such as the 'COM-B' system.

### Study limitations

Our systematic review has some limitations. First, despite our efforts to conduct an exhaustive search of the health databases, expanding the timeframes of the published studies (e.g., 2010 to the present) compared to other systematic literature reviews, and being as inclusive as possible when selecting ICT for managing frailty in older adults, we may have missed some that were not published or indexed in those databases. Second, conducting a meta-analysis and analyzing the quality of the evidence was impossible due to the diverse objectives of the designs and the outcome measures of the studies included in this systematic literature review. Finally, we did not conduct gray literature searches; as a result, some relevant reviews may not have been included.

In conclusion, the ICT used to manage frailty in older adults are not yet ready to be operated in an actual operating environment. Interventional studies on frailty using ICT were rarely reported. There is not much evidence that ICT are an effective intervention for delaying frailty. As a result, ICT are not yet ready to be a full-fledged technology for managing frailty in the older adult. population.

## Supplementary Materials

The Supplemenantry data can be found online at: www.aginganddisease.org/EN/10.14336/AD.2020.1114.


