# Supplementary material for: Information and Communication Technologies for Managing Frailty: A Systematic Literature Review
Source: Aging Dis. 2021 Jun 1;12(3):914–33. doi: 10.14336/AD.2020.1114 (PMC8139198; doi:10.14336/AD.2020.1114)
Supplement: Supplementary file 1 [file AD-12-3-914-s.pdf]

SUPPLEMENTARY DATA

# Information and Communication Technologies for Managing Frailty: A Systematic Literature Review

**Antonio Miguel Cruz[1,2,3*], Laura Monsalve[4], Anna-Maria Ladurner[1], Luisa Fernanda Jaime[4], Daniel Wang[1], Daniel Alejandro Quiroga[4]**

[1]Department of Occupational Therapy, Faculty of Rehabilitation Medicine, University of Alberta, Edmonton, AB, Canada. [2]Glenrose Rehabilitation Research, Innovation & Technology (GRRIT) Hub, Glenrose Rehabilitation Hospital, Edmonton, AB, Canada. [3]Faculty of Applied Health Sciences, University of Waterloo, Waterloo, ON, Canada. [4]School of Medicine and Health Sciences, Universidad del Rosario, Bogota, Colombia.

© 2020. Miguel Cruz A et al. Published online at http://www.aginganddisease.org/EN/10.14336/AD.2020.1114

# SUPPLEMENTARY DATA

**Supplementary Table 1.** Peer-reviewed Literature Search Strategy.

**Medline February 26, 2020.**

| # | Query | Results |
|---|---|---|
| 1 | exp Frailty/ | 2113 |
| 2 | (frail* or fragility).mp. [mp=title, abstract, original title, name of substance word, subject heading word, floating sub-heading word, keyword heading word, organism supplementary concept word, protocol supplementary concept word, rare disease supplementary concept word, unique identifier, synonyms] | 43515 |
| 3 | 1 or 2 | 43515 |
| 4 | exp Information Technology/ | 307 |
| 5 | exp Information Systems/ | 221651 |
| 6 | exp Technology/ | 406871 |
| 7 | exp Communication/ | 301525 |
| 8 | exp Computer Communication Networks/ | 90424 |
| 9 | exp Telecommunications/ | 89270 |
| 10 | exp Speech Recognition Software/ | 704 |
| 11 | exp Internet/ | 77093 |
| 12 | exp Internet Access/ | 36 |
| 13 | exp Telemedicine/ | 27196 |
| 14 | exp Telerehabilitation/ | 324 |
| 15 | exp Wearable Electronic Devices/ | 10927 |
| 16 | exp Wireless Technology/ | 3372 |
| 17 | exp Monitoring, Ambulatory/ | 28002 |
| 18 | exp Electronics, Medical/ | 6439 |
| 19 | exp Self-Help Devices/ | 11366 |
| 20 | exp Cell Phone/ | 10269 |
| 21 | exp Text Messaging/ | 2662 |
| 22 | exp Electronic Mail/ | 2641 |
| 23 | exp Computers/ | 77325 |
| 24 | exp Computers, Handheld/ | 7352 |
| 25 | exp Mobile Applications/ | 5334 |
| 26 | exp Cloud Computing/ | 564 |
| 27 | exp Software/ | 154078 |
| 28 | exp Computing Methodologies/ | 957184 |

© 2020. Miguel Cruz A et al. Published online at http://www.aginganddisease.org/EN/10.14336/AD.2020.1114

| | | |
|---|---|---|
| 29 | (Pedar-X or sensor*).mp. [mp=title, abstract, original title, name of substance word, subject heading word, floating sub-heading word, keyword heading word, organism supplementary concept word, protocol supplementary concept word, rare disease supplementary concept word, unique identifier, synonyms] | 389180 |
| 30 | (internet or world wide web or telecommunication* or (web-based not web-based survey*) or cloud-based).mp. [mp=title, abstract, original title, name of substance word, subject heading word, floating sub-heading word, keyword heading word, organism supplementary concept word, protocol supplementary concept word, rare disease supplementary concept word, unique identifier, synonyms] | 122798 |
| 31 | (information adj2 (technolog* or system*)).mp. [mp=title, abstract, original title, name of substance word, subject heading word, floating sub-heading word, keyword heading word, organism supplementary concept word, protocol supplementary concept word, rare disease supplementary concept word, unique identifier, synonyms] | 88721 |
| 32 | (cellphone* or smartphone* or ((cell* or mobile or smart) adj2 (phone* or telephone*)) or personal-digital assistant or personal-data-assistant* or pocket-pc*).mp. [mp=title, abstract, original title, name of substance word, subject heading word, floating sub-heading word, keyword heading word, organism supplementary concept word, protocol supplementary concept word, rare disease supplementary concept word, unique identifier, synonyms] | 25644 |
| 33 | ((Text* adj2 messag*) or short message service or SMS or texting).mp. [mp=title, abstract, original title, name of substance word, subject heading word, floating sub-heading word, keyword heading word, organism supplementary concept word, protocol supplementary concept word, rare disease supplementary concept word, unique identifier, synonyms] | 10110 |
| 34 | ((e-mail* not (e-mail*-survey or e-mail*-questionnaire*)) or (email* not (email-survey* or email*-questionnaire*)) or electronic-mail or electronic-messag*).mp. [mp=title, abstract, original title, name of substance word, subject heading word, floating sub-heading word, keyword heading word, organism supplementary concept word, protocol supplementary concept word, rare disease supplementary concept word, unique identifier, synonyms] | 16691 |
| 35 | (app or apps or applications or ((mobile or software) adj3 application)).mp. [mp=title, abstract, original title, name of substance word, subject heading word, floating sub-heading word, keyword heading word, organism supplementary concept word, protocol supplementary concept word, rare disease supplementary concept word, unique identifier, synonyms] | 482927 |
| 36 | ((mobile adj3 health) or mhealth or ehealth or m-health or e-health).mp. [mp=title, abstract, original title, name of substance word, subject heading word, floating sub-heading word, keyword heading word, organism supplementary concept word, protocol supplementary concept word, rare disease supplementary concept word, unique identifier, synonyms] | 15935 |
| 37 | (Telemedicine or tele-medicine or telehealth or tele-health or tele-monitor* or telemonitor* or tele-care or telecare or tele-consult* or teleconsult* or tele-therap* or teletherap* or telerehab* or tele-rehab* or e-rehab* or ((remote or virtual) adj4 rehab*)).mp. [mp=title, abstract, original title, name of substance word, subject heading word, floating sub-heading word, keyword heading word, organism supplementary concept word, protocol supplementary concept word, rare disease supplementary concept word, unique identifier, synonyms] | 35642 |
| 38 | ((remote* or video) adj3 (assess* or consult* or monitor*)).mp. [mp=title, abstract, original title, name of substance word, subject heading word, floating sub-heading word, keyword heading word, organism supplementary concept word, protocol supplementary concept word, rare disease supplementary concept word, unique identifier, synonyms] | 14258 |
| 39 | (((speech or voice) adj2 recognition) or Alexa or cortana or Siri or google-home or teach-back communication*).mp. [mp=title, abstract, original title, name of substance word, subject heading word, floating sub-heading word, keyword heading word, organism supplementary concept word, protocol supplementary concept word, rare disease supplementary concept word, unique identifier, synonyms] | 7080 |
| 40 | ((wearable or wireless or medical or assistive or self-help or portable) adj4 (device* or technolog* or electronic*)).mp. [mp=title, abstract, original title, name of substance word, subject heading word, floating sub-heading word, keyword heading word, organism supplementary concept word, protocol supplementary concept word, rare disease supplementary concept word, unique identifier, synonyms] | 75554 |

© 2020. Miguel Cruz A et al. Published online at http://www.aginganddisease.org/EN/10.14336/AD.2020.1114

| | | |
|---|---|---|
| 41 | ((device* or tool*) adj4 (Windows or Mac or LINUX or measur* or assess* or monitor*)).mp. [mp=title, abstract, original title, name of substance word, subject heading word, floating sub-heading word, keyword heading word, organism supplementary concept word, protocol supplementary concept word, rare disease supplementary concept word, unique identifier, synonyms] | 109297 |
| 42 | ((continuous or intermittent or physiological) adj3 (monitor* or track*)).mp. [mp=title, abstract, original title, name of substance word, subject heading word, floating sub-heading word, keyword heading word, organism supplementary concept word, protocol supplementary concept word, rare disease supplementary concept word, unique identifier, synonyms] | 23843 |
| 43 | (computer* or computing or software or hardware or laptop* or tablet* or ipad*).mp. [mp=title, abstract, original title, name of substance word, subject heading word, floating sub-heading word, keyword heading word, organism supplementary concept word, protocol supplementary concept word, rare disease supplementary concept word, unique identifier, synonyms] | 1035581 |
| 44 | 4 or 5 or 6 or 7 or 8 or 9 or 10 or 11 or 12 or 13 or 14 or 15 or 16 or 17 or 18 or 19 or 20 or 21 or 22 or 23 or 24 or 25 or 26 or 27 or 28 or 29 or 30 or 31 or 32 or 33 or 34 or 35 or 36 or 37 or 38 or 39 or 40 or 41 or 42 or 43 | 3050590 |
| 45 | 3 and 44 | 4585 |
| 46 | limit 45 to ("all child (0 to 18 years)" or "young adult (19 to 24 years)" or "adult (19 to 44 years)" or "young adult and adult (19-24 and 19-44)" or "middle age (45 to 64 years)" or "middle aged (45 plus years)") | 2849 |
| 47 | limit 46 to "all aged (65 and over)" | 2518 |
| 48 | 45 not (46 not 47) | 4254 |
| 49 | (osteoporosis or animals or heart or cancer or cells or fracture or bone or mineral).ti. | 2431842 |
| 50 | 48 not 49 | 3534 |
| 51 | limit 50 to yr="2010 -Current" | 2443 |

## Web of Science February 26, 2020

| # | Query | Results |
|---|---|---|
| 1 | TOPIC: (frail* or fragility) | 54,839 |
| 2 | TOPIC: (Pedar-X or sensor*)<br>*Indexes=SCI-EXPANDED, SSCI, A&HCI, CPCI-S, CPCI-SSH, BKCI-S, BKCI-SSH, ESCI, CCR-EXPANDED, IC Timespan=All years* | 1,114,669 |
| 3 | TOPIC: ((internet or world wide web or telecommunication* or (web-based not web-based survey*) or cloud-based))<br>*Indexes=SCI-EXPANDED, SSCI, A&HCI, CPCI-S, CPCI-SSH, BKCI-S, BKCI-SSH, ESCI, CCR-EXPANDED, IC Timespan=All years* | 397,222 |
| 4 | TOPIC: ((information NEAR/2 (technolog* or system*)))<br>*Indexes=SCI-EXPANDED, SSCI, A&HCI, CPCI-S, CPCI-SSH, BKCI-S, BKCI-SSH, ESCI, CCR-EXPANDED, IC Timespan=All years* | 284,337 |
| 5 | TOPIC: ((cellphone* or smartphone* or ((cell* or mobile or smart) NEAR/2 (phone* or telephone*)) or personal-digital assistant or personal-data-assistant* or pocket-pc*))<br>*Indexes=SCI-EXPANDED, SSCI, A&HCI, CPCI-S, CPCI-SSH, BKCI-S, BKCI-SSH, ESCI, CCR-EXPANDED, IC Timespan=All years* | 90015 |
| 6 | TOPIC: (((Text* NEAR/2 messag*) or short message service or SMS or texting))<br>*Indexes=SCI-EXPANDED, SSCI, A&HCI, CPCI-S, CPCI-SSH, BKCI-S, BKCI-SSH, ESCI, CCR-EXPANDED, IC Timespan=All years* | 22,407 |

© 2020. Miguel Cruz A et al. Published online at http://www.aginganddisease.org/EN/10.14336/AD.2020.1114

# SUPPLEMENTARY DATA

| | | |
|---|---|---|
| 7 | TOPIC: (((e-mail* not (e-mail*-survey or e-mail*-questionnaire*)) or (email* not (email-survey* or email*-questionnaire*)) or electronic-mail or electronic-messag*))<br>*Indexes=SCI-EXPANDED, SSCI, A&HCI, CPCI-S, CPCI-SSH, BKCI-S, BKCI-SSH, ESCI, CCR-EXPANDED, IC Timespan=All years* | 35,210 |
| 8 | TOPIC: ((app or apps or applications or ((mobile or software) NEAR/3 application)))<br>*Indexes=SCI-EXPANDED, SSCI, A&HCI, CPCI-S, CPCI-SSH, BKCI-S, BKCI-SSH, ESCI, CCR-EXPANDED, IC Timespan=All years* | 4,003,249 |
| 9 | TOPIC: (((mobile NEAR/3 health) or mhealth or ehealth or m-health or e-health))<br>*Indexes=SCI-EXPANDED, SSCI, A&HCI, CPCI-S, CPCI-SSH, BKCI-S, BKCI-SSH, ESCI, CCR-EXPANDED, IC Timespan=All years* | 20,320 |
| 10 | TOPIC: ((Telemedicine or tele-medicine or telehealth or tele-health or tele-monitor* or telemonitor* or tele-care or telecare or tele-consult* or teleconsult* or tele-therap* or teletherap* or telerehab* or tele-rehab* or e-rehab* or ((remote or virtual) NEAR/4 rehab*)))<br>*Indexes=SCI-EXPANDED, SSCI, A&HCI, CPCI-S, CPCI-SSH, BKCI-S, BKCI-SSH, ESCI, CCR-EXPANDED, IC Timespan=All years* | 29,340 |
| 11 | TOPIC: (((remote* or video) NEAR/3 (assess* or consult* or monitor*)))<br>*Indexes=SCI-EXPANDED, SSCI, A&HCI, CPCI-S, CPCI-SSH, BKCI-S, BKCI-SSH, ESCI, CCR-EXPANDED, IC Timespan=All years* | 31,409 |
| 12 | TOPIC: ((((speech or voice) NEAR/2 recognition) or Alexa or cortana or Siri or google-home or teach-back communication*))<br>*Indexes=SCI-EXPANDED, SSCI, A&HCI, CPCI-S, CPCI-SSH, BKCI-S, BKCI-SSH, ESCI, CCR-EXPANDED, IC Timespan=All years* | 34,708 |
| 13 | TOPIC: (((wearable or wireless or medical or assistive or self-help or portable) NEAR/4 (device* or technolog* or electronic*)))<br>*Indexes=SCI-EXPANDED, SSCI, A&HCI, CPCI-S, CPCI-SSH, BKCI-S, BKCI-SSH, ESCI, CCR-EXPANDED, IC Timespan=All years* | 131,727 |
| 14 | TOPIC: ((device* or tool*) NEAR/4 (Windows or Mac or LINUX or measur* or assess* or monitor*))<br>*Indexes=SCI-EXPANDED, SSCI, A&HCI, CPCI-S, CPCI-SSH, BKCI-S, BKCI-SSH, ESCI, CCR-EXPANDED, IC Timespan=All years* | 230,024 |
| 15 | TOPIC: (((continuous or intermittent or physiological) NEAR/3 (monitor* or track*)))<br>*Indexes=SCI-EXPANDED, SSCI, A&HCI, CPCI-S, CPCI-SSH, BKCI-S, BKCI-SSH, ESCI, CCR-EXPANDED, IC Timespan=All years* | 41,879 |
| 16 | TOPIC: ((computer* or computing or software or hardware or laptop* or tablet* or ipad*))<br>*Indexes=SCI-EXPANDED, SSCI, A&HCI, CPCI-S, CPCI-SSH, BKCI-S, BKCI-SSH, ESCI, CCR-EXPANDED, IC Timespan=All years* | 2,663,234 |
| 17 | #16 OR #15 OR #14 OR #13 OR #12 OR #11 OR #10 OR #9 OR #8 OR #7 OR #6 OR #5 OR #4 OR #3 OR #2<br>*Indexes=SCI-EXPANDED, SSCI, A&HCI, CPCI-S, CPCI-SSH, BKCI-S, BKCI-SSH, ESCI, CCR-EXPANDED, IC Timespan=All years* | 7,772,872 |
| 18 | #17 AND #1<br>*Indexes=SCI-EXPANDED, SSCI, A&HCI, CPCI-S, CPCI-SSH, BKCI-S, BKCI-SSH, ESCI, CCR-EXPANDED, IC Timespan=All years* | 7,458 |
| 19 | TI=((child* or adolescent* or teen* or "young adult*" or youth* or toddler*))<br>*Indexes=SCI-EXPANDED, SSCI, A&HCI, CPCI-S, CPCI-SSH, BKCI-S, BKCI-SSH, ESCI, CCR-EXPANDED, IC Timespan=All years* | 1,198,394 |
| 20 | #18 NOT #19<br>*Indexes=SCI-EXPANDED, SSCI, A&HCI, CPCI-S, CPCI-SSH, BKCI-S, BKCI-SSH, ESCI, CCR-EXPANDED, IC Timespan=All years* | 7,393 |
| 21 | TI=(osteoporosis OR animals OR heart OR cancer OR cells OR fracture OR bone OR mineral)<br>*Indexes=SCI-EXPANDED, SSCI, A&HCI, CPCI-S, CPCI-SSH, BKCI-S, BKCI-SSH, ESCI, CCR-EXPANDED, IC Timespan=All years* | 4,830,024 |
| 22 | #20 NOT #21<br>*Indexes=SCI-EXPANDED, SSCI, A&HCI, CPCI-S, CPCI-SSH, BKCI-S, BKCI-SSH, ESCI, CCR-EXPANDED, IC Timespan=2010-2020* | 4640 |

© 2020. Miguel Cruz A et al. Published online at http://www.aginganddisease.org/EN/10.14336/AD.2020.1114

# SUPPLEMENTARY DATA

## EMBASE February 26, 2020

| # | Query | Results |
|---|---|---|
| 1 | exp frailty/ | 9353 |
| 2 | (frail* or fragility).mp. [mp=title, abstract, heading word, drug trade name, original title, device manufacturer, drug manufacturer, device trade name, keyword, floating subheading word, candidate term word] | 72985 |
| 3 | 1 or 2 | 72985 |
| 4 | exp automatic speech recognition/ | 1028 |
| 5 | exp sensor/ | 103262 |
| 6 | exp wireless communication/ | 5081 |
| 7 | exp smartphone/ | 11151 |
| 8 | exp mobile phone/ | 26864 |
| 9 | exp personal digital assistant/ | 1418 |
| 10 | exp tablet computer/ | 881 |
| 11 | exp text messaging/ | 4664 |
| 12 | exp mobile application/ | 10628 |
| 13 | exp assistive technology device/ | 907 |
| 14 | exp assistive technology/ | 1752 |
| 15 | (Pedar-X or sensor*).mp. [mp=title, abstract, heading word, drug trade name, original title, device manufacturer, drug manufacturer, device trade name, keyword, floating subheading word, candidate term word] | 490150 |
| 16 | (internet or world wide web or telecommunication* or (web-based not web-based survey*) or cloud-based).mp. [mp=title, abstract, heading word, drug trade name, original title, device manufacturer, drug manufacturer, device trade name, keyword, floating subheading word, candidate term word] | 179829 |
| 17 | (information adj2 (technolog* or system*)).mp. [mp=title, abstract, heading word, drug trade name, original title, device manufacturer, drug manufacturer, device trade name, keyword, floating subheading word, candidate term word] | 128311 |
| 18 | (cellphone* or smartphone* or ((cell* or mobile or smart) adj2 (phone* or telephone*)) or personal-digital assistant or personal-data-assistant* or pocket-pc*).mp. [mp=title, abstract, heading word, drug trade name, original title, device manufacturer, drug manufacturer, device trade name, keyword, floating subheading word, candidate term word] | 37371 |
| 19 | ((Text* adj2 messag*) or short message service or SMS or texting).mp. [mp=title, abstract, heading word, drug trade name, original title, device manufacturer, drug manufacturer, device trade name, keyword, floating subheading word, candidate term word] | 14532 |
| 20 | ((e-mail* not (e-mail*-survey or e-mail*-questionnaire*)) or (email* not (email-survey* or email*-questionnaire*)) or electronic-mail or electronic-messag*).mp. [mp=title, abstract, heading word, drug trade name, original title, device manufacturer, drug manufacturer, device trade name, keyword, floating subheading word, candidate term word] | 36536 |

© 2020. Miguel Cruz A et al. Published online at http://www.aginganddisease.org/EN/10.14336/AD.2020.1114

# SUPPLEMENTARY DATA

| | | |
|---|---|---|
| 21 | (app or apps or applications or ((mobile or software) adj3 application)).mp. [mp=title, abstract, heading word, drug trade name, original title, device manufacturer, drug manufacturer, device trade name, keyword, floating subheading word, candidate term word] | 522691 |
| 22 | ((mobile adj3 health) or mhealth or ehealth or m-health or e-health).mp. [mp=title, abstract, heading word, drug trade name, original title, device manufacturer, drug manufacturer, device trade name, keyword, floating subheading word, candidate term word] | 14189 |
| 23 | (Telemedicine or tele-medicine or telehealth or tele-health or tele-monitor* or telemonitor* or tele-care or telecare or tele-consult* or teleconsult* or tele-therap* or teletherap* or telerehab* or tele-rehab* or e-rehab* or ((remote or virtual) adj4 rehab*)).mp. [mp=title, abstract, heading word, drug trade name, original title, device manufacturer, drug manufacturer, device trade name, keyword, floating subheading word, candidate term word] | 47087 |
| 24 | ((remote* or video) adj3 (assess* or consult* or monitor*)).mp. [mp=title, abstract, heading word, drug trade name, original title, device manufacturer, drug manufacturer, device trade name, keyword, floating subheading word, candidate term word] | 16207 |
| 25 | (((speech or voice) adj2 recognition) or Alexa or cortana or Siri or google-home or teach-back communication*).mp. [mp=title, abstract, heading word, drug trade name, original title, device manufacturer, drug manufacturer, device trade name, keyword, floating subheading word, candidate term word] | 9233 |
| 26 | ((wearable or wireless or medical or assistive or self-help or portable) adj4 (device* or technolog* or electronic*)).mp. [mp=title, abstract, heading word, drug trade name, original title, device manufacturer, drug manufacturer, device trade name, keyword, floating subheading word, candidate term word] | 182727 |
| 27 | ((device* or tool*) adj4 (Windows or Mac or LINUX or measur* or assess* or monitor*)).mp. [mp=title, abstract, heading word, drug trade name, original title, device manufacturer, drug manufacturer, device trade name, keyword, floating subheading word, candidate term word] | 178413 |
| 28 | ((continuous or intermittent or physiological) adj3 (monitor* or track*)).mp. [mp=title, abstract, heading word, drug trade name, original title, device manufacturer, drug manufacturer, device trade name, keyword, floating subheading word, candidate term word] | 35956 |
| 29 | (computer* or computing or software or hardware or laptop* or tablet* or ipad*).mp. [mp=title, abstract, heading word, drug trade name, original title, device manufacturer, drug manufacturer, device trade name, keyword, floating subheading word, candidate term word] | 1883358 |
| 30 | 4 or 5 or 6 or 7 or 8 or 9 or 10 or 11 or 12 or 13 or 14 or 15 or 16 or 17 or 18 or 19 or 20 or 21 or 22 or 23 or 24 or 25 or 26 or 27 or 28 or 29 | 3378282 |
| 31 | 3 and 30 | 9511 |
| 32 | limit 31 to (child or adult <18 to 64 years>) | 2947 |
| 33 | limit 32 to aged <65+ years> | 1265 |
| 34 | 31 not (32 not 33) | 7829 |
| 35 | (osteoporosis or animals or heart or cancer or cells or fracture or bone or mineral).ti. | 2983106 |
| 36 | 34 not 35 | 5245 |
| 37 | limit 36 to yr="2010 -Current" | 4169 |

© 2020. Miguel Cruz A et al. Published online at http://www.aginganddisease.org/EN/10.14336/AD.2020.1114

# SUPPLEMENTARY DATA

## CINAHL February 26, 2020

| # | Query | Results |
|---|---|---|
| S1 | Frailty Syndrome | 2,636 |
| S2 | frail* or fragility | 20,450 |
| S3 | Communication | 189,688 |
| S4 | Wireless Communications | 12,326 |
| S5 | Communication Skills | 25,637 |
| S6 | Communications Media | 15,554 |
| S7 | Telecommunications | 3,164 |
| S8 | Technology | 161,027 |
| S9 | Assistive Technology Services | 1,970 |
| S10 | Assistive Technology | 11,186 |
| S11 | Information Technology | 44,282 |
| S12 | Assistive Technology Devices | 7,221 |
| S13 | Wearable Sensors | 2,253 |
| S14 | Voice Recognition Systems | 1,466 |
| S15 | Computing Methodologies | 600 |
| S16 | Information Systems | 94,028 |
| S17 | Computer Communication Networks | 3,628 |
| S18 | Internet | 66,224 |
| S19 | Telemedicine | 18,231 |
| S20 | Telerehabilitation | 519 |
| S21 | Telehealth | 18,134 |
| S22 | Electrical Equipment and Supplies | 1,750 |
| S23 | Cellular Phone | 2,156 |
| S24 | Text Messaging | 3,667 |
| S25 | Instant Messaging | 487 |
| S26 | Email | 13,518 |
| S27 | Telephone | 40,843 |
| S28 | Computers, Portable | 2,626 |
| S29 | Computers, Hand-Held | 4,643 |
| S30 | Computers and Computerization | 10,912 |

© 2020. Miguel Cruz A et al. Published online at http://www.aginganddisease.org/EN/10.14336/AD.2020.1114

# SUPPLEMENTARY DATA

| | | |
|---|---|---|
| S31 | Mobile Applications | 9,988 |
| S32 | Simulation | 49,613 |
| S33 | Pedar-X or sensor* | 52,032 |
| S34 | (internet or world wide web or telecommunication* or (web-based not web-based survey*) or cloud-based) | 148,648 |
| S35 | (information N2 (technolog* or system*)) | 60,323 |
| S36 | (cellphone* or smartphone* or ((cell* or mobile or smart) N2 (phone* or telephone*)) or personal-digital assistant or personal-data-assistant* or pocket-pc*) | 16,604 |
| S37 | ((Text* N2 messag*) or short message service or SMS or texting) | 5,771 |
| S38 | ((e-mail* not (e-mail*-survey or e-mail*-questionnaire*)) or (email* not (email-survey* or email*-questiolectronic-mail or electronic-mennaire*)) or essag*) | 14,018 |
| S39 | (app or apps or applications or ((mobile or software) N3 application)) | 152,193 |
| S40 | ((mobile N3 health) or mhealth or ehealth or m-health or e-health) | 20,606 |
| S41 | (Telemedicine or tele-medicine or telehealth or tele-health or tele-monitor* or telemonitor* or tele-care or telecare or tele-consult* or teleconsult* or tele-therap* or teletherap* or telerehab* or tele-rehab* or e-rehab* or ((remote or virtual) N4 rehab*)) | 23,484 |
| S42 | ((remote* or video) N3 (assess* or consult* or monitor*)) | 5,716 |
| S43 | (((speech or voice) N2 recognition) or Alexa or cortana or Siri or google-home or teach-back communication*) | 5,627 |
| S44 | ((wearable or wireless or medical or assistive or self-help or portable) N4 (device* or technolog* or electronic*)) | 101,011 |
| S45 | (device* or tool*) N4 (Windows or Mac or LINUX or measur* or assess* or monitor*) | 199,726 |
| S46 | ((continuous or intermittent or physiological) N3 (monitor* or track*)) | 6,331 |
| S47 | (computer* or computing or software or hardware or laptop* or tablet* or ipad*) | 559,326 |
| S48 | S3 OR S4 OR S5 OR S6 OR S7 OR S8 OR S9 OR S10 OR S11 OR S12 OR S13 OR S14 OR S15 OR S16 OR S17 OR S18 OR S19 OR S20 OR S21 OR S22 OR S23 OR S24 OR S25 OR S26 OR S27 OR S28 OR S29 OR S30 OR S31 OR S32 OR S33 OR S34 OR S35 OR S36 OR S37 OR S38 OR S39 OR S40 OR S41 OR S42 OR S43 OR S44 OR S45 OR S46 OR S47 | 1,378,686 |
| S49 | S1 OR S2 | 20,450 |
| S50 | S48 AND S49 | 5,124 |
| S51 | S48 AND S49 | 1,232 |
| S52 | S51 | 949 |
| S53 | S50 not (S51 not S52) | 4,841 |
| S54 | TI(osteoporosis OR animals OR heart OR cancer OR cells OR fracture OR bone OR mineral) | 452,268 |
| S55 | S53 not S54 | 2,815 |

© 2020. Miguel Cruz A et al. Published online at http://www.aginganddisease.org/EN/10.14336/AD.2020.1114

# SUPPLEMENTARY DATA

**Supplementary Table 2.** Levels of evidence (According to Teasell's approach (Available from: https://pdfs.semanticscholar.org/2b08/beaff788b361f6e3f5ca37ed8fda616b3fa9.pdf.))

| Level of evidence | Description |
|---|---|
| Level 1a | More than one randomized controlled trial with PEDro scores ≥ 6. Includes within subject's comparison with randomized conditions and cross-over designs |
| Level 1b | One randomized controlled trial with a PEDro score ≥ 6. Includes within subject's comparison with randomized conditions and cross-over designs. |
| Level 2 | Randomized controlled trial, PEDro score < 6; Non-RCTS and Cohort studies (using at least 2 similar groups with one exposed to a particular condition). |
| Level 3 | Case-Control: A retrospective study comparing conditions, including historical controls. |
| Level 4 | Case Series, retrospective chart review; Pre-Post, a prospective trial with a baseline measure, intervention, and a post-test using a single group of subjects; or Post-Study, a prospective post-test with two or more groups, intervention, then post-test using a single group of subjects |
| Level 5 | Observational, studies using cross-sectional analysis to interpret relations; Clinical Consensus, expert opinion without explicit critical appraisal, or based on physiology, biomechanics or "first principles"; or Case Report, pre-post or case studies (n=1). |
| Conflicting | In the absence of evidence, agreement by a group of experts on the appropriate treatment course. Consensus opinion is regarded as the lowest form of evidence. As such, it is arguably not considered evidence at all. Disagreement between the findings of at least 2 RCTs or where RCTs are not available. Where there are more than 4 RCTs and the results of only one was conflicting, the conclusion was based on the results of the majority of the studies, unless the study with conflicting results was of higher quality. |

© 2020. Miguel Cruz A et al. Published online at http://www.aginganddisease.org/EN/10.14336/AD.2020.1114
